# Comparison of Flows through a Tidal Inlet in Late Spring and after the Passage of an Atmospheric Cold Front in Winter Using Acoustic Doppler Profilers and Vessel-Based Observations

**DOI:** 10.3390/s22093478

**Published:** 2022-05-03

**Authors:** Mingming Li, Chunyan Li

**Affiliations:** 1 Laboratory for Coastal Ocean Variation and Disaster Prediction, College of Ocean and Meteorology, Guangdong Ocean University, Zhanjiang 524088, China; limm@gdou.edu.cn; 2 Key Laboratory of Climate, Resources and Environment in Continental Shelf Sea and Deep Sea of Department of Education of Guangdong Province, Guangdong Ocean University, Zhanjiang 524088, China; 3 Key Laboratory of Space Ocean Remote Sensing and Application, Ministry of Natural Resources, Beijing 100081, China; 4 Department of Oceanography and Coastal Sciences, Louisiana State University, Baton Rouge, LA 70803, USA; 5 Coastal Studies Institute, Louisiana State University, Baton Rouge, LA 70803, USA

**Keywords:** acoustic sensor, ADCP, hydrodynamic measurements, weather impact, atmospheric cold front, flow structures, inverse estuary, wind-driven circulation

## Abstract

This paper discusses the application of acoustic Doppler current profilers (ADCP) for the quantification of transport of water and the underlining physical mechanism. The transport of water through estuaries and tidal inlets is affected by tide, river flow, and wind. It is often assumed that wind effects in such systems are negligible unless under severe weather conditions. This study compares the ADCP-measured flows across a tidal inlet under weak wind conditions in late spring and those after the passage of an atmospheric cold front in winter. The Barataria Pass is a major inlet connecting Barataria Bay and northern Gulf of Mexico. The water exchange between the bay and coastal ocean is influenced by wind, especially in winter, because tide in the region is small (microtidal). The winter weather and late spring–summer weather are different. This difference results in different estuarine circulations. To examine this, two surveys were carried out with ship-mounted ADCPs—one in winter (19 December 2014) shortly after the passage of a cold front from the northwest, and the other in late spring (4 May 2015) with weak southeasterly winds. Distinctly different features of mean transport through the inlet were observed between the two surveys. The results from the first survey in winter showed that the total water transport was from the bay to the coastal ocean under northerly winds with intense outflows in shallow water, which is a typical signature of wind effects. The net flow was outward when the water level dropped. Data from the second survey in spring showed that the mid-channel water flew out of the bay (against the wind), whilst inflow appeared at both ends across the inlet, which was also a response to the weak wind stress and outward pressure gradient force set by the estuarine flow. The inflow at the eastern end (exceeding 0.1 m/s) is consistent with the idea that the coastal current resulted from the Mississippi River outflow enters the bay from the eastern end. The influence of tidal oscillations on water exchange appeared to be higher in the late spring data. The hydrographic observations in spring showed typical tidal straining features of an inverse estuary during the ebb–flood cycle, while salinity in the eastern shallow water generally varied with time, indicating the inflow of fresher water into the bay, confirming previous observations from summer 2008.

## 1. Introduction

Sensors measuring current velocity in fluids have evolved over time. The simplest mechanical current meters are the predecessors of the many modern-day state-of-the-art sensors. Measurements of flow in the environment and in industrial applications, such as in a pipe, are also very different. In industrial applications, the device is often called a flowmeter, which can be based on the measurements of volume flux, mass flux, pressure differences, or other dynamic parameters or proxies, and can be mechanical, acoustic, electromagnetic, or optical [1,2,3]. In hydrodynamic applications, including those in the ocean, mechanical propeller current meters have been used for decades, e.g., rotary current meters or RCMs [4,5,6]. Since ocean water is salty and a good conductor of electricity, electromagnetic current meters were once widely used in coastal ocean observations [7]. From the 1980s onwards, acoustic Doppler current profilers [8] started to replace and even dominate the sensors used for in situ velocity measurements. The main reason for this shift was that the acoustic sensors are quasi-remote sensing tools; they can provide a profile of velocity values, rather than just a single point measurement. This is equivalent to having a series of sensors placed along the direction of acoustic beams sent from the sensor unit or transducer. As a result, the velocity profiles are obtained instantaneously and non-intrusively, providing an efficient and reliable method of measurement.

**Figure 1:**
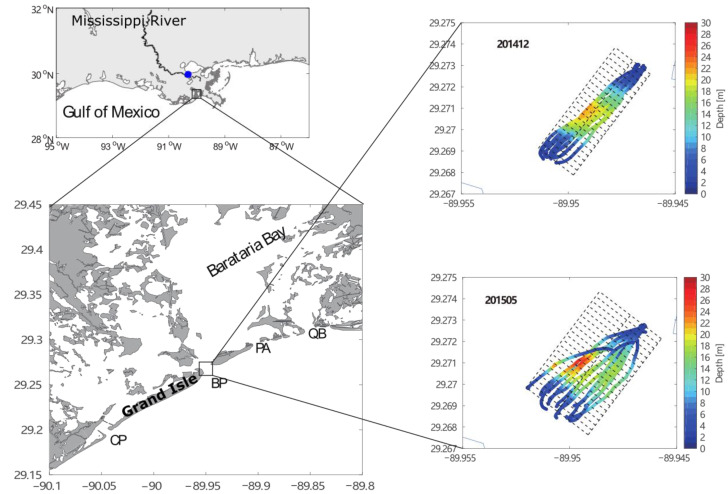
Map of study area and two section tracks from investigations in December 2014 and May 2015. The blue dot on the upper left panel marks the location of New Orleans Airport, where the wind field was observed. The colors in the right panels indicate the water depths measured along the track. The four inlets labeled in the lower left panel are the Caminada Pass (CP), Barataria Pass (BP), Pass Abel (PA), and Quatre Bayou Pass (QB) from southwest to northeast.

In this study, we apply such acoustic sensors for measurements of the flow velocity field across a tidal inlet in Louisiana, USA. The Louisiana (LA) coast in the northern Gulf of Mexico (GOM) is dominated by a diurnal microtide with a maximal tidal range of ~0.6 m [9]. Barataria Bay (BB) is located in southeast LA, with the Mississippi River Birdfoot Delta to the east. It is shallow, with a mean depth of about 2 m. The horizontal scale of the semi-enclosed bay is about 40 km × 35 km. There are four major inlets at the southern boundary of BB, which are separated by several barrier islands and are the primary connections to the open water of the GOM (Figure 1). The bay is surrounded by swamp forests and marshes, meaning the study of this bay system is fundamental to the understanding of the local ecosystem [10,11]. The major inlets connecting the bay and the shelf include the Caminada Pass (CP), Barataria Pass (BP), Pass Abel (PA), and Quatre Bayou Pass (QB) from west to east (Figure 1). The BP between the Grand Isle and Grand Terre Island is the deepest among the four inlets but is relatively narrow (20 m deep and 800 m wide). There is a circular shaped deep depression of around 50 m inside the inlet of the BP according to previous observations [12,13], indicating strong velocity-induced erosion near the inlet. The salinity inside the bay varies significantly from season to season. The maximum salinity can reach 27 the at surface and ~29 near the bottom, ranging higher in summer and dropping down to 7–9 or lower in spring. The low salinity in spring is mainly due to the strong spring flooding from the Mississippi River [13].

The main processes that affect the exchange of estuarine water with the coastal ocean include tidal oscillations, wind, and river discharge [14,15,16]. To distinguish the main influencing factor and examine the features of the current field, measurements should cover at least a 24 h period. The transport can be calculated by measurements of velocity at fixed points with a single point current meter [17,18], vertical acoustic Doppler current profilers (ADCP) [15], or horizontal ADCPs [13]. These measurements can provide long time series and accordingly more reliable analysis of subtidal transport features at specific locations. However, due to the limitations of the instrument, the transport across the entire inlet can only be deduced through interpolation, making it less accurate. The use of ship-mounted ADCP measurements across the entire transect is another approach that has been widely used [19,20,21,22]. Vessel-based ADCPs can record the vertical profiles of currents from the surface via a series of vertical bins as the vessel moves along a predesigned route. The instantaneous transport across the transect can be derived via the integration of the velocity recordings from each bin along the transect. With repeated tracks of the same route, one can obtain the temporal variation of the transport along the transect, and consequently derive the tidal and subtidal components of transport. This method can be challenging when the current is swift and weather conditions are complicated. Conducting such a continuous survey is more challenging in a system with diurnal tides, as the measurements should last longer than in a system dominated by semidiurnal tides. A survey length of 24 h is preferred, although the harmonic analysis can still produce usable tidal constituents if the period is a little shorter than 24 h because the tidal frequencies are given. There is only a limited number of such surveys in this system to illustrate the cross-channel flow structure and responses to weather and wind [12].

Previous studies in Barataria Bay suggested that the flow through the main inlets of the bay is strongly influenced by wind, particularly during severe weather events such as in winter [13], and is more influenced by the tide in summer [12], unless during hurricanes. During cold fronts in winter, significant transport out of the bay (more than 40% of the total volume) onto the continental shelf may occur across time scales of 1–2 days [23]. These studies, however, did not examine the flow structure across the inlets and lacked a comparison with a more detailed analysis.

The major objective of this work is to compare the differences through direct observations in the flow structure between two seasons, winter and late spring, which have different weather and wind conditions. The hypothesis is that the water exchange patterns during these seasons are different because the winds under different weather conditions are different, meaning the relative importance of tides is different. Here, we specifically examine the bay–shelf water exchange through a major inlet of Barataria Bay, the Barataria Pass, in winter and late spring with ship-mounted ADCP measurements. We investigate the influencing factors on tidal and subtidal exchange flows through the inlet. The observations are described in the following section, together with the methods used in this study. The results are presented in Section 3, contrasting the two conditions during the two surveys. A discussion and summary are provided in the last two sections.

## 2. Materials and Methods

In this study, a SonTek M9 multi-frequency acoustic Doppler current profiler (ADCP) was used to measure current velocity profiles and to estimate the total transport across the Barataria Pass. The M9 ADCP is a 9-transducer Janus-type ADCP with a 25° slant angle. Four of the transducers work at 3.0 MHz frequency and four at 1.0 MHz, while the last one is a vertical transducer with a 500 KHz working frequency used as an echosounder to measure the water depth. The ADCP samples at a high ping rate of 70 Hz. The depth range of the M9 ADCP is between 0.2 and 80 m, with an accuracy level of ~1% of the total water depth and a resolution of 1 mm, while the velocity and discharge have a vertical range of 0.3–40 m with bottom tracking enabled or 0.3–80 m in navigation mode using differential GPS. The velocity range is ± 20 m/s, with an accuracy of ~0.25% of the measured velocity and a resolution of 0.001 m/s. The maximum number of vertical bins is 128 and the cell sizes range between 0.02 m and 4 m, being automatically adjusted according to the actual water depth.

Two surveys were conducted. The first survey was carried out in winter from 1455 UTC on 18 December to 1450 UTC on 19 December 2014, while the second survey was done in late spring from 1553 UTC on 3 May to 1615 UTC on 4 May 2015. To examine the tidal and subtidal components of the flow and transport, the observations should be a diurnal tidal cycle or longer. During the second survey, the GPS malfunctioned at the latter stage of observations, meaning the usable records ended at 1225 UTC on 4 May 2015 and the duration of the second investigation was only 20.5 h. This still allowed a harmonic analysis, since the diurnal and semi-diurnal tidal frequencies were provided.

During each survey, the M9 ADCP was mounted in front of a 24 ft research vessel (integrated with a GPS) with the transducers facing downward. The boat ran repeatedly across the ~800 m wide main channel, the Barataria Pass, along a predesigned section for about one diurnal tidal cycle. The maximal boat speed was less than 3 m/s to diminish bias in the velocity measurements. Bottom tracking was used to enhance the quality of the velocity measurements. The vertical bins varied from 0.06 m to 2 m from the shallowest to deepest water. The temporal ensemble interval of samplings was set to 1s in both cruises. The ADCP measured the velocity profiles and computed the transport internally.

The sections of the two observations were consistent but varied slightly (Figure 1), as the boat tracks were partly affected by the changing currents. The lengths of both sections were around 650 m. The boat avoided reaching water shallower than ~2 m. Both investigations were accompanied by CTD casts near the central deep water and at the east end, measuring the corresponding physical properties of water at various tidal phases. The CTD was a Seabird Electronics (SBE) 19 plus instrument, with a 4 Hz sampling rate.

Furthermore, we also used the water level records from a tidal gauge at Grand Isle, LA (station ID: 8761724), and wind field observations from New Orleans Airport (USW00012916, National Centers for Environmental Information, or NCEI, of NOAA) for analysis of relevant weather conditions. The water level data were downloaded from NOAA’s Center for Operational Oceanographic Products and Services (CO-OPS, [24]) and the wind data from the Global Historical Climatology Network (GHCN)—Daily Database of NOAA [25]. The temporal resolution of the water level and wind field was 6 min.

There were 14 repetitions of transects with ADCP records during each of the surveys. The duration of each track was between 4 and 9 min, and the ship speed was generally lower than 3 m/s. With these records of short duration with long gaps, we treated the records in every repetition of the transect as simultaneous measurements. The repeated ship tracks inevitably deviated from the predesigned sections due to the inaccuracy of navigation locations and the influence of winds, currents, and waves. The tracks of the two investigations are shown in Figure 1 with colored dots, which are colored based on the water depths derived from the 500 KHz transducer. One can notice the variation in vessel tracks, especially during the second investigation. This is typical of boat-based surveys. To analyze the data, we adopted an optimized harmonic analysis based on a gridding method, as suggested by [12]. All data points were bounded in a rectangular area measuring ~630 m × 250 m, with the longer sides being parallel to the designed track. Here, the points at both ends with sparse records were excluded to improve the accuracy of analyses. We divided the central axis of all tracks (central dashed thick lines in the northeast–southwest direction in Figure 1) into small segments of 30 m long and the entire area was separated into 21 subregions by the lines perpendicular to the central axis. All data points in the same subregion were assigned to the central point of the segment. Thus, we obtained time series for 21 selected data points. For the second cruise, the 650-m-long section was divided into 20 groups of 30-m-long segments and the sparse points were also excluded. One can see that the water depths varied along the whole section but were relatively uniform in each segment because the transect was roughly perpendicular to the main channel. Thus, the assignment to individual points did not induce significant error within each 30 m segment.

The ADCP data were first processed to exclude abnormally large values and misaligned records during vessel veering, passing of sharply varied topography, and due to sporadic outliers. The records were then averaged into 5 s intervals to further smooth the fluctuations and separated into segments, as shown in Figure 1. Since the vertical bin sizes varied according to the water depth, data points in the same segment were interpolated to the unified vertical levels. We then rotated the coordinates counterclockwise by 52.7° and projected the velocity vectors into the along-channel and cross-channel velocity components, with the flow into the bay being defined as the positive along-channel velocity. The focus of this study was to quantify tidal and subtidal flow fields through the inlet for both surveys, meaning only flows along the channel were analyzed. The total numbers of temporal records for each segment varied between 37 and 50 for the winter data and between 32 and 54 for the late spring data. We then applied harmonic analysis to the along-channel velocity for each segment of the dataset. Since the overall records during both investigations were relatively short, we could not distinguish different tidal constituents with close frequencies, so we chose the O1 and M2 constituents to represent the diurnal and semidiurnal tides, respectively. The wind vector used in this work was first treated with a 40 h low-pass filter to exclude high-frequency variations [26].

## 3. Results

### 3.1. Tidal and Subtidal Flows in Winter

**Figure 2:**
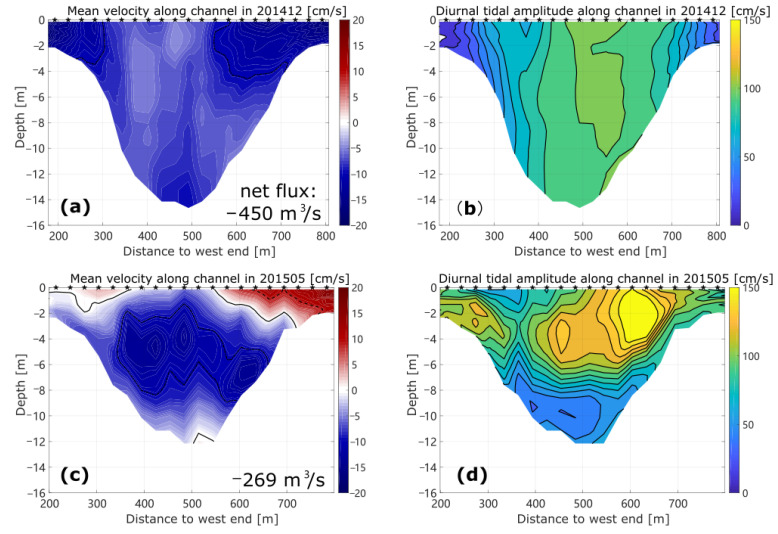
Subtidal and tidal current velocities in the along-channel direction during investigations in December 2014 (**a**,**b**) and May 2015 (**c**,**d**). The dotted, solid, and dashed lines (**a**,**c**) represent −10, 0, and 10 cm/s, respectively, while the sketched intervals (**b**,**d**) both represent 10 cm/s. The net transport estimated from the mean velocity along the channel are labeled (**a**,**c**), with negative signs indicating outward flow from the bay.

**Figure 3:**
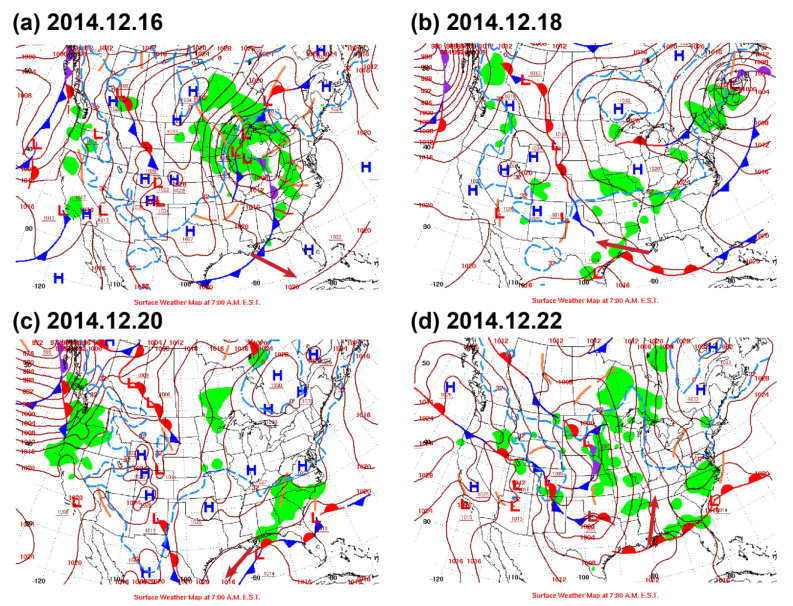
Daily surface weather maps at sea level around the survey time in December 2014 (downloaded from [35]). The red lines with numbers in figure are isobars; H and L indicate central areas of high pressure and low pressure, respectively; blue lines with triangles indicate the locations of cold fronts; red lines with semicircles indicate warm fronts; lines with alternate symbols on other sides are stationary fronts; purple lines with triangles and semicircles on the same side represent occlusions; green shading areas indicate precipitation regions; the red arrow stemming from our study area on each panel indicates the local wind direction at the study site. (**a**–**d**) Studies on the 16, 18, 20, and 22 December in the year 2014.

**Figure 4:**
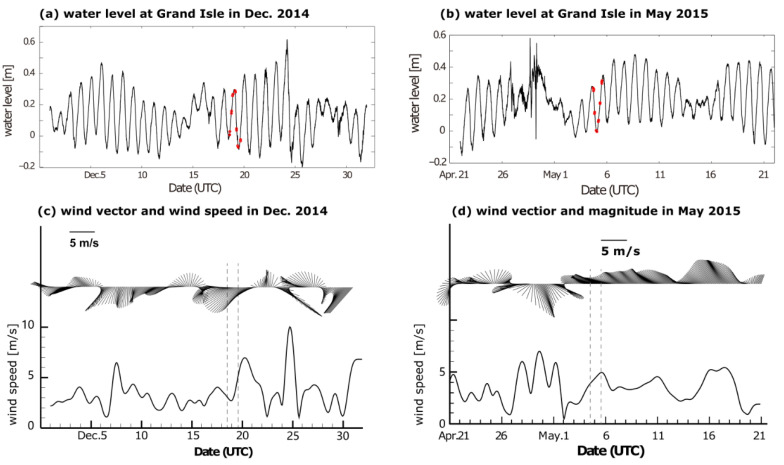
Water levels (**a**,**b**) and wind vectors (**c**,**d**) prior, during, and after ADCP investigations. The veering bars in lower panels indicate 40 h lower passed wind vectors with 2 h interval and the curves below show the wind magnitudes. The time of each track is labeled by red dots in both water level records, while the time durations are bounded by vertical dashed lines in corresponding wind fields.

The mean or de-tided velocity along the channel in the winter survey is illustrated in Figure 2a, where the bluish color indicates the outflow from the Barataria Bay. The overall subtidal net transport is from the BB to the northern gulf, and the magnitude of along-channel velocity is of the order of 10 cm/s, with the maximum values exceeding 15 cm/s in shallow waters and in the surface layer. The relatively greater values in shallow water are consistent with the effect of local northeasterly wind stress. The winter observation was carried out shortly after the passage of a cold front (Figure 3a) from northwest to southeast and prior to the northward propagation of a warm front in the northwest of the gulf (Figure 3d), i.e., during the northeasterly winds, as shown in the upper left panel of Figure 4. The northeasterly measured from New Orleans Airport is consistent with an overall outward flow and stronger flow over the shoals. As discussed in [27], the shallow water in broad lagoon and estuarine systems has flow in the same direction of the local wind, while the deeper water flows at slow speeds or even against the wind. This has been recognized through theoretical or numerical modeling [28,29,30] and observed in many systems [27,31,32,33].

**Figure 5:**
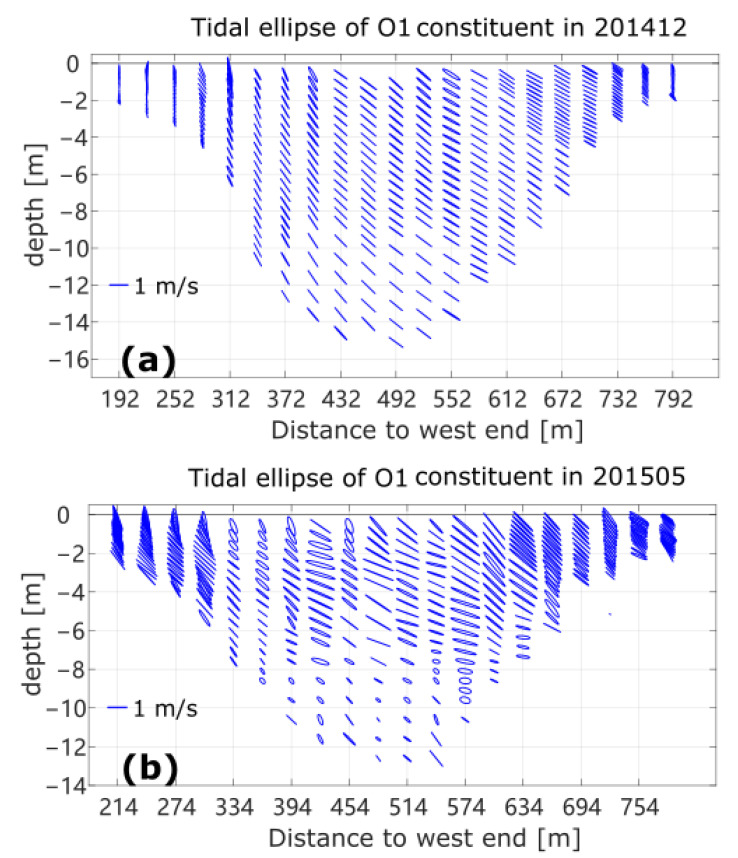
Tidal ellipse of diurnal components in winter observation (**a**) and late spring observation (**b**). The major and minor axes of current ellipses are isometric and shown in north and east directions and the blue bars in lower-left corner are scales of 1 m/s.

The contour of the diurnal tidal current velocity amplitude from the harmonic analysis of the along-channel velocity is shown in Figure 2b. The amplitude of the diurnal tidal component in the along-channel direction is generally smaller than 1 m/s during this survey. The large values located mainly in the middle columns of the section and those at the shallow ends are smaller than 30 cm/s. The survey time was during the transition from neap to spring tide (Figure 4a). This flow structure is consistent with the typical tidal flow amplitude across a channel in which the bottom friction is an important factor in the momentum balance [12,34]. The horizontal current ellipses of diurnal tidal component O1 during this survey are shown in Figure 5a. Compared with the alignment of the BP inlet, we can see that the diurnal tidal current generally flows back and forth in the along-channel direction, while the amplitude of tidal current decreases with the decrease in water depth. The amplitudes of the semidiurnal tidal current velocity are generally smaller than one-fifth of those of the diurnal component, although this is not shown. The astronomical tides mainly influence the deep water of the Barataria Pass and contribute to a large extent to the variation in flow across the BP inlet—about 85% of the flow variability in the pass was tidally induced, with equal contributions from the O1 and K1 constituents according to [15]. In our study, a 24 h time series was too short to separate O1 from K1 constituents (the minimum time required to separate the two frequencies is about 13.6 days). We can consider that the diurnal tidal component represented by O1 here is a combination of all diurnal tidal components.

### 3.2. Tidal and Subtidal Flows in Spring

Compared with the results from the winter survey, the distribution of mean velocity values along the channel of the BP inlet shows more variability in May 2015 (Figure 2c). This was expected because the winter survey was during a period of stronger winds after the passage of a cold front (Figure 4). The overall flow shown here is out of the bay, with a maximal subtidal velocity of over 0.1 m/s, but there is a relatively higher reversed flow (exceeding 10 cm/s) into the bay at the eastern shallow end and relatively weak inflow near the surface at the western end. The mean inward transport near the surface is consistent with the dominating southeasterly wind. Moreover, the intense outflow is concentrated in the mid-depth of the middle column. The inward flow in the shallow water in the eastern end is consistent with the research in [12], in which low-salinity water was shown to be flowing into the Barataria Basin along the east end of the inlet. It was suggested that the inflow is a mixture of the gulf water and the discharge of the Mississippi River, which may enter Barataria Bay, unless the wind is a northerly. The influence of the tidal component is higher in the late spring dataset. The amplitude of the diurnal constituent above 7 m water depth is mostly larger than 1 m/s (Figure 2d). The maximal amplitude is 1.7 m/s, centered at around 3 m to the east of middle column, with a minimal value of 0.4 m/s. The investigation was conducted from one flood to the next flood period between the neap and spring tides (tracked by the red dots in Figure 4b). The dominating local winds shown in Figure 4d are southeasterly and easterly, which are favorable for an inward flow of water from the coastal current with relatively lower salinity [12].

### 3.3. Hydrological Features during Observations

**Figure 6:**
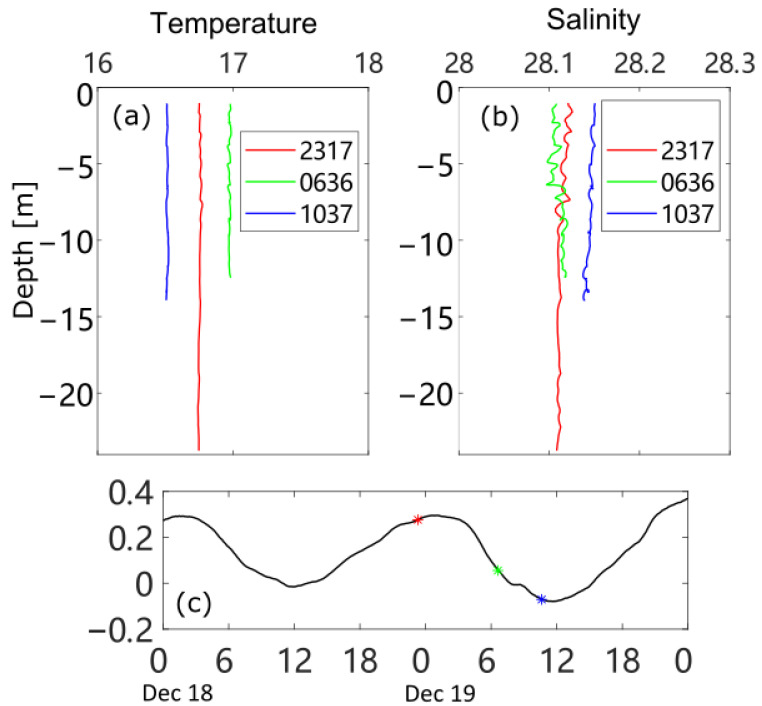
Temperature (**a**) and salinity (**b**) casts during the investigation in December 2014, and water levels labeled with times of corresponding casts (**c**).

Several CTD casts were carried out during both investigations. Three CTD casts in the winter observation were made in the middle of the channel, taken around the flood slack, in the middle of the ebb, and at the ebb slack (Figure 6). The whole water column is generally well-mixed, as shown by the nearly vertical profiles of temperature and salinity. The salinity at the ebb slack is slightly higher, indicating that the outflow water from the Barataria Bay is more saline in winter (an inverse estuarine condition, or a “salt plug” condition in this system, as studied in [36]).

**Figure 7:**
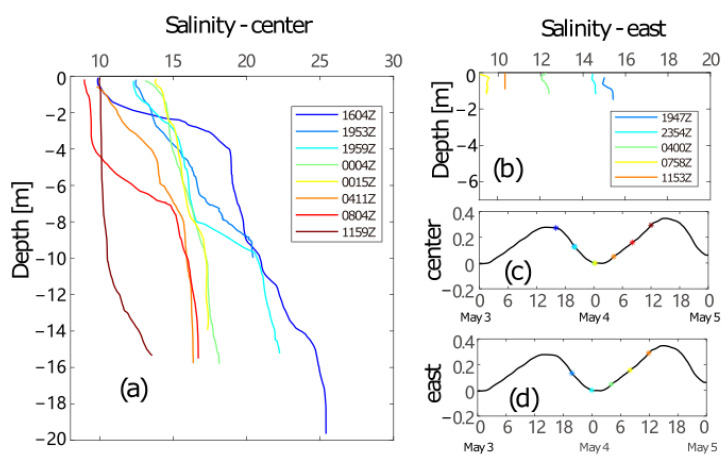
Salinity casts during the investigation in May 2015 in central deep water (**a**) and eastern shallow water (**b**), and water levels labeled with times of central casts (**c**) and eastern casts (**d**).

Temperature and salinity measurements were also carried out during the spring investigation, mainly conducted in the central deep water and eastern part of the section with shallow water. The temperature records show limited variabilities, and we will just focus on the vertical salinity profiles in this work. All casts are separated here into 2 groups for clarity: one for the deep casts in the middle of the section (Figure 7a) and the other for the shallow casts near the eastern end (Figure 7b). The times the casts are marked on the water level records by colored asterisks (Figure 7c,d) corresponding to different casts. The eastern shallow water was well-mixed throughout the survey, and the salinity decreased during the entire period, with a subtle increase at the end of the flood. Combined with the subtidal (mean) inward flow and relatively small amplitude of the tidal ellipse (Figure 5), we could conclude that there was an inflow of relatively lower-salinity water from the coast at the eastern end during the observation, consistent with the summer conditions [37], but differing from the winter conditions.

The variation in salinity in the central section shows an inverse estuarine feature of tidal straining [37,38,39]. The central column was highly stratified in the beginning of the observations (around flood slack stage), with a vertical salinity difference of approximately 15 PSU and an apparent halocline of between 2 and 5 m (cast 1604Z). The stratification subsequently weakened during the following ebb period. Salinity near the surface increased continuously from 10 PSU to around 14 PSU shortly before the ebb slack, and at 14 m decreased from around 24 PSU at the first cast to 17.5 PSU. Meanwhile, the halocline deepened to 8–10 m four hours later (cast 1959Z) and disappeared shortly before the ebb slack (cast 0004Z and 0015Z). The whole column was well mixed at the end of the ebb tide and the vertical salinity difference was only 4 PSU. The stratification increased during the following flood period. The surface salinity decreased to 9 PSU in the middle of the flood, and the strong gradient emerged again at 4–7 m with a salinity difference of 6 PSU (cast 0804Z). The salinity at 14 m was still smaller than all casts during the ebb tide. The observation was carried out during the severe spring flooding of the Mississippi River, with plenty of freshwater spreading along the LA coast, meaning the coastal water was fresher than that in Barataria Bay. Fresher water entered the bay during the flood and established strong stratification of the flood slack. Then, as the saltier water flushed out of the bay, the salinity of the surface water increased and tended to destruct the stratification. The whole column was then mixed vertically at the ebb slack. On the contrary to conventional tidal straining [38], the hydrological observation of this work showed a clear inverse estuary feature. In comparison, in [38], the tidal straining showed asymmetric characteristics between the western and eastern ends in summer, also indicating an inverse estuarine process, at least at the eastern end.

## 4. Discussion

### 4.1. The Influence of Wind vs. Tidal Cycle

As shown in the preceding section, the net flow across the Barataria Pass is influenced by both wind stress and astronomical tides, but with varying responses depending on the location and season. In general, the tidal cycle dominates in the deeper central inlet, while the wind stress has a more intense impact at the shallow ends.

The strength of the influence from the tidal cycle is clearly illustrated by the major axis of the tidal ellipse in Figure 5. The amplitude of the diurnal tidal constituent exceeds 1 m/s in the whole column of the central water in winter. The decay and veering of the current of the lower central water in spring should be the combined action of the bottom friction and wind stress. The tidal flows in the western and eastern shallow ends diminish to below 0.2 m/s in winter due to the bottom friction, but are much stronger in late spring, mostly in the along-channel direction. The inward flow with favorable wind stress in late spring tends to force more offshore water towards the channel, consequently enhancing the flow from tides across shallow inlets.

The wind influences the net transport of more salient water in winter. Since the winter observations were conducted just after the passage of a cold front, severe northerly and northeasterly winds drove the whole water column out of the bay, with faster flow in shallower waters at both ends. The net transport driven by wind is much more subtle in spring. Although the winds before and during the measurements were favored inward flow, the residual current in the central part was out of the bay, with a slightly weakened inward flow near the central bottom area. This may have been due to freshwater-discharge-induced net outward flow. The maximum discharge from the head of the bay is estimated to be 250 m^3^/s [12].

### 4.2. Tidal Straining

The inward net flow appears in shallow waters, carrying fresh Mississippi River flood water into the bay at the eastern end, and consequently forming inverse estuarine tidal straining, consistent with [37] in summer.

The temporal variation of salinity in late spring measurements reveals a clear cycle of tidal straining, although with an inverse estuarine feature. Tidal currents in estuaries often display vertical shearing of the horizontal velocity due to bottom friction, thereby producing unstable conditions during the flood tide and promoting vertical mixing to reach a well-mixed condition. In a normal estuary, the water is vertically stratified during the ebb tide, since fresh estuary water moves faster in the upper layer and suppresses vertical mixing [37,38]. The vertical salinity structure in the late spring reveals an opposite profile: well-mixed water appears during the late ebb stage and shortly after the transition from ebb to flood stages (yellow, green, and orange lines in Figure 7a), while more stable water can be found at the flood slack area and following the early ebb phase. The inverse estuary mode is mainly formed due to the dilution of coastal water by later spring flooding of the Mississippi River [37]. The maximal salinity of the central inlet is smaller than that observed in winter. The well-mixed shallow water in the eastern end is also covered by relatively fresh water during the flood tide, confirming the invasion of low-salinity water from the coast.

## 5. Concluding Remarks

We carried out two hydrodynamic surveys across one of the major inlets in Barataria Bay on the northern coast of the Gulf of Mexico to investigate the tidal and subtidal transport features in winter and late spring. The velocity data were obtained using acoustic sensors (ADCPs). The main findings were as follows:

(1) In microtidal systems such as Barataria Bay, the impact of weather is more important than that of the tidally driven flow (the temporally averaged currents induced by tides), especially in winter;
(2) In this region, the winter weather is very different compared to late spring and summer weather —it is dominated by atmospheric cold front passages at 3–7 day intervals, which keeps the water column vertically well mixed and the water level oscillation and related flushing of the bays enhanced;
(3) The winter and late spring flow structures are consistent with the wind conditions in these seasons, with net outflow in winter forced by strong northerly winds and a two-layered flow structure in late spring, with inflow in shallow water and the upper layer caused by weak–medium southeasterly winds and outflow in deep water by the pressure gradient force;
(4) The vertical structure of salinity in the central BP pass confirms an inverse estuarine condition in late spring; the stratification is strongest at the flood slack when fresher coastal water occupies the upper water layer and diminishes as the coastal water retreats;
(5) The low-salinity coastal water may flow into the bay in late spring; the southeasterly wind in late spring drives the coastal water, which is diluted by the Mississippi River discharge due to spring flooding, into the eastern end of the BP and spreads westward in the channel, whereas the northerly wind after cold front passage in winter impedes coastal water from entering the bay;
(6) As far as vessel-based transect surveys are concerned, this work complements previous studies in this area, which only focused on summer conditions across the transect (except moored observations at fixed locations and numerical experiments). This work shows that the winter and late spring conditions have certain similarities: the along-channel transport responds to weather conditions; and diurnal velocity amplitudes decrease with water depth.

The use of ADCPs also made this work efficient compared to conventional mechanical RCMs [18], which would require much more manpower and effort to obtain data with comparable coverage in space and time but with much lower resolutions. Although there have been many applications of vessel-based surveys with ADCPs in other systems, this study provides a comparison of the transport in different seasons under different weather conditions in this system. Future studies should examine the overall impacts of the global weather system [40] on local extreme weather conditions and evaluate in more detail how much influence severe weather events can have on coastal ocean and estuaries.

## Data Availability

Data for this paper were collected by C.L. and can be accessed at https://oceandynamics.lsu.edu/data/barataria.htm (accessed on 20 March 2022).
